# Ectomycorrhizal identification in environmental samples of tree roots by Fourier-transform infrared (FTIR) spectroscopy

**DOI:** 10.3389/fpls.2014.00229

**Published:** 2014-05-27

**Authors:** Rodica Pena, Christa Lang, Annette Naumann, Andrea Polle

**Affiliations:** Forest Botany and Tree Physiology, Büsgen-Institut, Georg-August University GöttingenGöttingen, Germany

**Keywords:** soilborne fungi, mycorrhiza, deciduous forests, infrared spectroscopy, cluster analysis, field samples

## Abstract

Roots of forest trees are associated with various ectomycorrhizal (ECM) fungal species that are involved in nutrient exchange between host plant and the soil compartment. The identification of ECM fungi in small environmental samples is difficult. The present study tested the feasibility of attenuated total reflection Fourier-transform infrared (ATR-FTIR) spectroscopy followed by hierarchical cluster analysis (HCA) to discriminate *in situ* collected ECM fungal species. Root tips colonized by distinct ECM fungal species, i.e., *Amanita rubescens*, *Cenococcum geophilum*, *Lactarius subdulcis*, *Russula ochroleuca*, and *Xerocomus pruinatus* were collected in mono-specific beech (*Fagus sylvatica*) and mixed deciduous forests in different geographic areas to investigate the environmental variability of the ECM FTIR signatures. A clear HCA discrimination was obtained for ECM fungal species independent of individual provenance. Environmental variability neither limited the discrimination between fungal species nor provided sufficient resolution to discern species sub-clusters for different sites. However, the de-convoluted FTIR spectra contained site-related spectral information for fungi with wide nutrient ranges, but not for *Lactarius subdulcis*, a fungus residing only in the litter layer. Specific markers for distinct ECM were identified in spectral regions associated with carbohydrates (i.e., mannans), lipids, and secondary protein structures. The present results support that FTIR spectroscopy coupled with multivariate analysis is a reliable and fast method to identify ECM fungal species in minute environmental samples. Moreover, our data suggest that the FTIR spectral signatures contain information on physiological and functional traits of ECM fungi.

## Introduction

Soilborne ectomycorrhizal fungi live in symbiotic associations with the roots of woody species (Smith and Read, 2008). By colonization, the fungus enwraps the entire surface of the root tip with a multi-layered hyphal mantle, forms a hyphal network in the extracellular compartment between adjacent cells of the root cortex, and spreads hyphae into the soil forming an external mycelium (Agerer, 2001). This novel composite organ involves structural and metabolic participation of both organisms (Smith and Read, 2008), the plant and the fungus, and is called ectomycorrhiza (ECM). ECMs improve host plant nutrition, especially under unfavorable environmental conditions (Pena and Polle, 2014), and increase plant stress tolerance (Schützendübel and Polle, 2002). They are important in main ecosystem processes such as carbon cycling and nutrient mobilization because they link plant and soil (Finlay, 2008). In forests, trees are usually colonized by a large number of different ECM fungal species (Buée et al., 2005; Pena et al., 2010; Lang et al., 2011). However, often the community composition remains enigmatic because of the difficulty in identifying fungal species *in situ*, in their vegetative state as ECM (Suz et al., 2008).

Typically, identification of the fungal partner in the ECM is carried out by morphological and anatomical microscopic inspections, followed by sequencing of the internal transcribed spacer region (ITS) of the colonized root tips. The microscopic observations are time-consuming and need highly skilled personal since the morphology of distinct ECM species can vary with fungal age and is often similar among different species (Agerer, 1987), while the molecular methods furthermore require the availability of well-equipped laboratory facilities. It would therefore be desirable to have methods that allow high-through put fungal identification with minimum sample preparation for minute amounts of tissue. In this study we investigate the suitability and limits of Fourier transform infrared (FTIR) spectroscopy for the distinction of ECMs in field samples.

FTIR spectroscopy has already been successfully applied to detect and identify fungi (Galichet et al., 2001; Mohaček-Grošev et al., 2001; Erukhimovitch et al., 2005; Naumann et al., 2005; Naumann, 2009). However, in these studies, the fungi were cultivated under strictly controlled growth conditions to avoid alterations of the FTIR spectra due to fungal responses to environmental changes. Consequently, the variation of the chemical fingerprints was entirely due to the inherent attributes of the fungal taxa under identical milieu conditions. For example, wood-inhabiting and degrading fungi were distinguished by FTIR fingerprinting (Erukhimovitch et al., 2005; Naumann et al., 2005; Linker and Tsror Lahkim, 2008). In a pioneering study, Calderon et al. (2009) showed that FTIR spectroscopy could be used to discriminate between arbuscular mycorrhizal and non-mycorrhizal root cultures. However, studies that distinguish the symbiotic organisms *in situ* are currently lacking.

The primary goal of this study was to test the feasibility of FTIR spectroscopy to discriminate ECMs formed with different fungal species in environmental samples. We compared the FTIR spectra of root tips colonized by distinct ECM collected across different scales from different forest types, stands and soil core samples. The sites were located in mono-specific beech (*Fagus sylvatica*) and mixed deciduous forests (Dannenmann et al., 2009; Leuschner et al., 2009). The study sites differ in leaf litter input into the soil, microbial biomass, and mineralization rates and nutrient concentrations in the roots of different tree species (Guckland et al., 2010; Jacob et al., 2010; Lang and Polle, 2011). We hypothesized that (i) ECM fungal species within a given site are discriminated by FTIR spectroscopy and multivariate data analysis and (ii) environmental differences result in biochemical differences in ECMs that are reflected in the spectra, thereby, overriding species-specific differences. To test these hypotheses, we characterized the variation in the FTIR spectra for five ECM species (*Amanita rubescens*, *Cenococcum geophilum*, *Lactarius subdulcis*, *Russula ochroleuca*, *Xerocomus pruinatus*), which are often found in European forest stands (Pena et al., 2010; Lang and Polle, 2011).

## Materials and methods

### Site description and sample collection

The study sites are located in different forest types in the National Park Hainich, Thuringia, Germany (51°05′28″N, 10°31′ 24″E, 350 m above sea level). The mean annual temperature is 7.5°C, and the mean annual precipitation is 670 mm (Leuschner et al., 2009). The soil profiles are classified as a Luvisol derived from Triassic limestone covered with 60-70 cm loess (World Reference Base for Soil Resources, 2006).

Further study sites are located in an old-growth beech forest located in the low mountain range of the Swabian Jura, in southwest Germany (47°59′N, 8°45′E, 800 m a.s.l.), with an average annual mean temperature of 6.5°C and a mean annual precipitation of 854 mm (for details see Dannenmann et al., 2009; Simon et al., 2011). The soil belongs to Rendzic Leptosols derived from limestone and marls with high fractions of stones and rocks (World Reference Base for Soil Resources, 2006).

Samples were collected in spring 2007 in three beech dominated forest (two in the Hainich with 95% beech, one in the Swabian Jura with 90% beech) and in four stands of various deciduous tree mixtures where beech represented 10 to 45%. The tree mixtures contained ash (*Fraxinus excelsior*), lime (*Tilia* sp.), hornbeam (*Carpinus betulus*), and maple (*Acer* sp.). Details of forest site soil characteristics are given in Table 1. The soil in beech stands was more acidic, and leaf litter contained less nitrogen than in the mixed stands (Guckland et al., 2009). The samples were collected by extracting 10 to 15 (Hainich) and 7 (Swabian Alb) soil cores (*r* = 40 mm, depth = 200 mm) per site. The soil samples were stored in polyethylene bags at 4°C until processing.

**Table 1:** **Soil characteristics (0–10 cm) and ectomycorrhizal samples collected in the mono-specific beech forest (Mono) and in the mixed (Mixed) deciduous forest stands in the National Park Hainich and in a beech forest in the Swabian Alb**.

**Fungal species**	**Hainich**		**Swabian Alb**
	**Mono**	**Mixed**	
pH	5.0	6.3	6.8
C/N ratio	17.2	14.2	13.2
Soil texture (sand/silt/clay)	3/81/16	2/73/26	3/26/70
Soil water content (vol%)	24.12	23.48	nd
**Fungal species**	**Number of ECM samples**		
*Amanita rubescens*	7	10^*^	0
*Cenococcum geophilum*	4	40^**^	17
*Lactarius subdulcis*	32	35^***^	0
*Russula ochroleuca*	13	0	0
*Xerocomus pruinatus*	21	2	0

Soil data were obtained from the following sources: pH from Mölder et al. (2008), C/N ratio from Guckland et al. (2009), soil water content from Krämer and Hölscher (2010), and data for Swabian Jura forest from Dannenmann et al. (2007).

nd, data not available.

* 5 with hornbeam roots.

** 8 with lime roots, 3 with hornbeam roots.

*** 2 with lime roots.

### Collection and identification of ECM root tips

The fine roots, harvested after washing the soil, were used for morphotyping as previously described (Pena et al., 2010; Lang et al., 2011). Root tips colonized by *Amanita rubescens*, *Cenococcum geophilum*, *Lactarius subdulcis*, *Russula ochroleuca*, and *Xerocomus pruinatus* were collected for FTIR spectroscopic measurements and ITS rDNA sequencing (for details see Lang et al., 2011). The sequences were deposited in NCBI GenBank with the accession numbers: EU346870; EU346872; EU346875; EU350580; EU350582.

A phylogenetic tree of nucleotide sequences alignments for the ITS regions was computed by MEGA5 software (Tamura et al., 2011). Phylogenies were inferred by the Neighbor–Joining method (Saitou and Nei, 1987). The evolutionary distances were computed using the Maximum Composite Likelihood method (Tamura and Kumar, 2002) and are given in units of the number of base substitutions per site.

For FTIR analyses, ECM root tips were excised at the level of last lateral root ramification, enwrapped by the fungal mantle. In total, 61 samples were collected for *Cenococcum geophilum*, 67 for *Lactarius subdulcis*, 13 for *Russula ochroleuca*, 23 for *Boletus pruinatus*, and 17 for *Amanita rubescens* ECMs (Table 1). The samples were stored at −80°C, freeze- dried and kept in a desiccator at room temperature for further spectroscopic measurements.

### FTIR spectra acquisition and analysis

Infrared spectra of ECM root tips, cellulose (Sigma-Aldrich^®^ C-6413, Munich, Germany), ergosterol (Sigma-Aldrich^®^ 45480, Munich, Germany) and chitin isolate from crab shells (Carl Roth^®^ 8845, Karlsruhe, Germany) were measured in the wavenumber range from 4000 to 600 cm^−1^ with an ATR unit (DuraSampl*IR*, Sens*IR* Europe, Warrington, UK) combined with an FTIR spectrometer (Equinox 55, Brucker Optics, Ettlingen, Germany) at a resolution of 4 cm^−1^ and 32 scans per sample. The root tips were pressed against the ATR crystal in such a way that the entire diamond crystal was covered. Giving the small dimensions of one root tip and the large morphological differences among species, we used two to five root tips for one measurement.

Evaluation and processing of the spectral data were performed using the software OPUS 6.5 (Brucker Optics, Ettlingen, Germany).

To identify the peaks in the spectra, we used the *Peak Picking* command in OPUS software. To be independent of the baseline, the second derivative, obtained by Savitzky-Golay algorithm, with 17 smoothing points was used.

Fourier self-deconvolution (FSD) was applied to enhance the spectral resolution and to obtain the position of the overlapping components in the selected spectral range for ECM species comparisons.

To explore the similarity patterns between spectral samples, we applied hierarchical cluster analysis (HCA) to the second derivative vector normalized FTIR spectra with 17 smoothing points using the spectral windows corresponding to the absorbance of fungal characteristic chemical functional groups (Förster et al., 2003): (I) fatty acids 3000–2800 cm^−1^; (II) amide I 1700–1600 cm^−1^; (III) amide II and III 1575–1300 cm^−1^; (IV) polysaccharides 1200–900 cm^−1^; (V) the “fingerprint region” (900–600 cm^−1^), which consists of a variety of weak, but very characteristic, spectral features. To calculate the spectral distance values necessary for HCA dendrograms construction, Euclidian distance and Ward's minimum variance were employed. The spectral differences were displayed as heterogeneity.

## Results

### ATR-FTIR spectroscopy yields information on the outer fungal mantle

**Figure 1:**
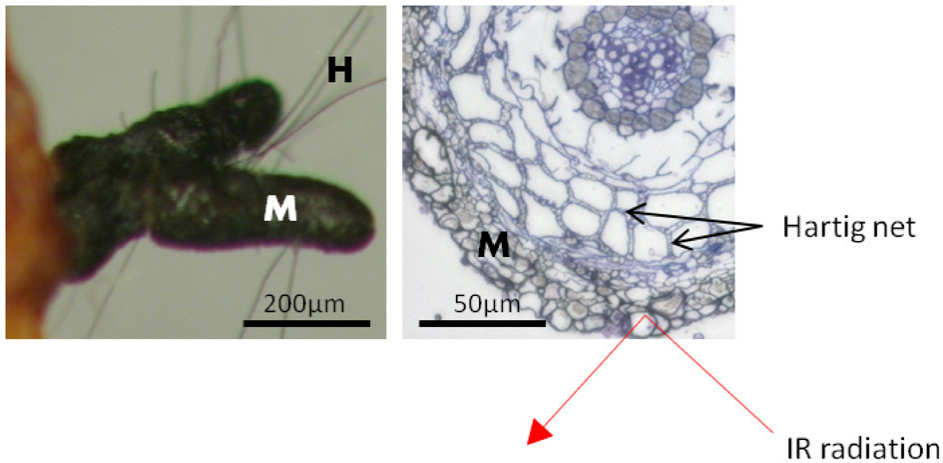
**Scheme of a beech root tip colonized by the ectomycorrhizal fungus *Cenoccocum geophilum*.** The fungus enwraps the root tip with a hyphal mantle [M] composed of several layers. The Hartig net is formed by fungal hyphae between cortex cells. Short hyphae [H] extend from the mantle into the surroundings. The penetration depth of the infrared radiation (IR), of about 2 μm, is limited to the outer layer of the fungal mantle. Scale bar: 50 μm.

The fungal mantle, by which the ectomycorrhizal fungi enwrapped the root tips, varied in thickness and structure with fungal identity (Table S1). Yet, in all ectomycorrhizae, it comprised several layers of hyphae with cell diameters of 2.5 to 20 μm (Table S1). Since ATR is a surface measuring technique to a maximum depth of 2 μm (Griffiths and Haseth, 2007), the acquired IR absorption profiles exclusively allowed interrogation of the very last hyphal layer of the outer ECM fungal mantle (Figure 1). The lack of spectral bands corresponding to the characteristic bands of cellulose at 1641 cm^−1^, 1427 cm^−1^, 1335 cm^−1^, 1160 cm^−1^ (Wilson et al., 2000; Naumann et al., 2010) confirmed the absence of plant contributions to the spectral yield.

### Hierarchical clustering resulted in 97% discrimination between ECM forming fungal species

**Figure 2:**
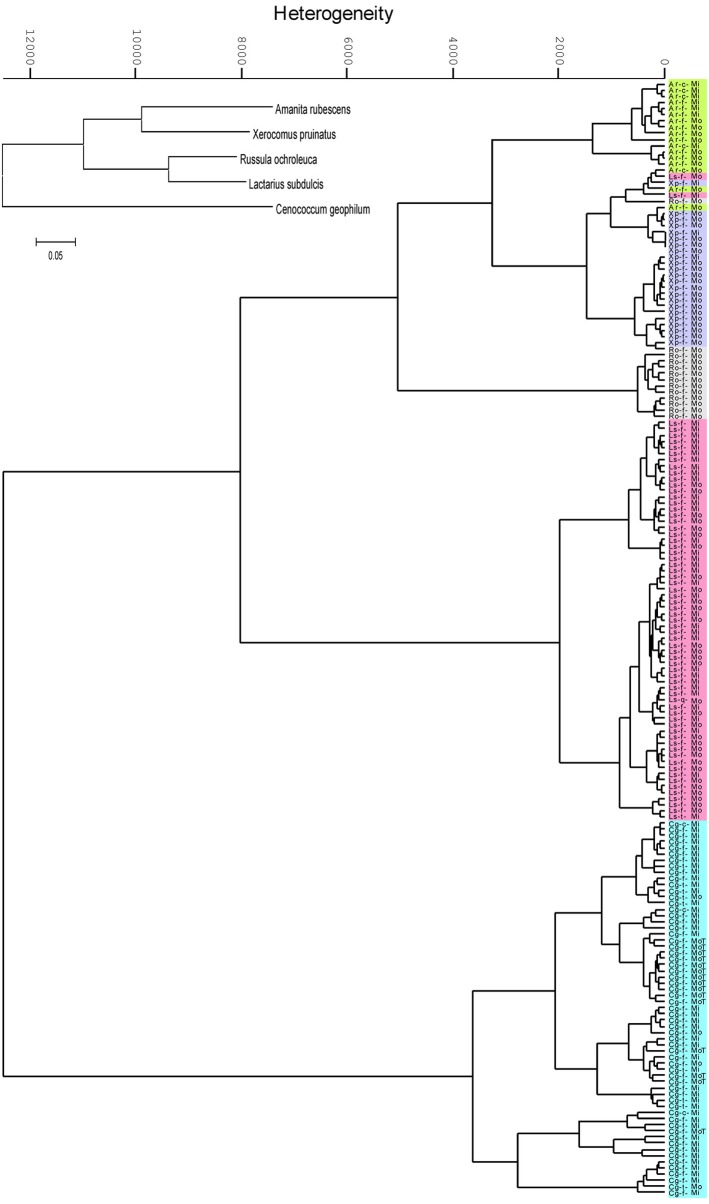
**Dendrogram of the second derivative of ectomycorrhizal FTIR spectra, clustered by Ward's algorithm in the range between the wavenumbers from 3000 to 2800 and from 1700 to 600 cm^−1^.** Ectomycorrhizas of beech (f), lime (t) and hornbeam (c) with *Amanita rubescens* (Ar, green), *Xerocomus pruinatus* (Xp, mauve), *Russula ochroleuca* (Ro, grey), *Lactarius subdulcis* (Ls, pink) or *Cenoccocum geophilum* (Cg, turquoise) collected in mono-specific beech forest stands (Mo) and in mixed deciduous forests (Mi). The inset shows the phylogenetic relationships of the fungal taxa.

Hierarchical cluster analysis (HCA) based on the absorbance properties of the major chemical constituents of the ECMs resulted in formation of two main clusters supporting the phylogenetic classification at the phylum level: the Basidiomycota cluster including *Amanita*, *Xerocomus*, *Russula* and *Lactarius* species, and the Ascomycota cluster with *Cenococcum geophilum* (Figure 2). However, the phylogenetic relationships were not anymore present at the family level since the Basidiomycota group was split in two sub-clusters. The spectral similarity between *Lactarius* and *Russula*, both belonging to *Russulaceae* family, was by a factor of 1.5 lower than the similarity between *Russula* and the *Amanita* - *Xerocomus* group (Figure 2), whereas phylogenetically they were clustered together (inset Figure 2). Each fungal species formed a unique cluster within the HCA dendogram, where all but six of 181 spectra were correctly assigned to their species group (Figure 2). Interestingly, the six incorrectly assigned spectra formed a distinct sub-cluster within the group of *Xerocomus pruinatus* (Figure 2).

The most striking result of the HCA was that environmental variations did not hinder the correct assignment of fungal species. The spectral heterogeneity within the individual species clusters exceeded the heterogeneity between samples collected in beech-dominated and mixed forests (Figure 2). Therefore, within a species group, samples from mono-specific and mixed forests could not be discriminated. In order to further explore the effect of environmental conditions on the suitability of IR spectroscopy to discriminate between ECM species, 15 samples of *C. geophilum* collected in beech forest on the Swabian Jura were included in the analysis; all of them were clustered within the *C. geophilum* species group. However, 60% of these samples formed an own sub-cluster, which exhibited a 7.8 times lower heterogeneity than the species cluster, indicating a moderate environment-spectral alteration compared with that given by fungal species identity (Figure 2).

Several samples in our analysis represented ECMs formed with different tree species (Table 1), but this had no consequences on fungal mantle biochemical composition (Figure 2).

### ECM forming fungi showed a species-specific spectral shape

To compare mean ECM spectra the most significant wavenumbers for the polysaccharide, lipid and protein compounds were interpreted.

**Figure 3:**
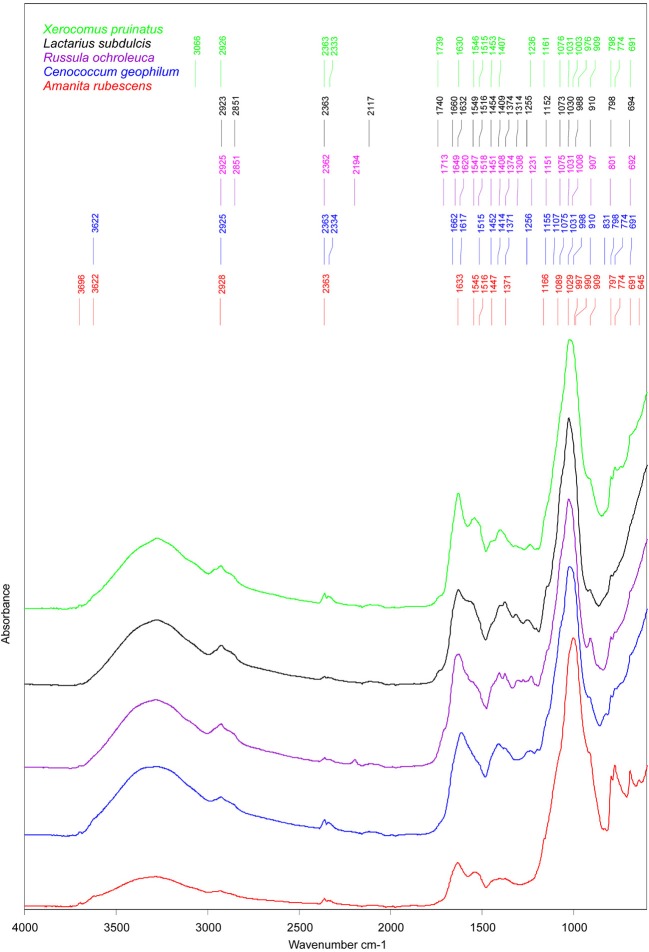
**Mean FTIR spectra of field-collected ectomycorrhizas formed with *Amanita rubescens* (*n* = 17), *Xerocomus pruinatus* (*n* = 23), *Russula ochroleuca* (*n* = 13), *Lactarius subdulcis* (*n* = 67), and *Cenoccocum geophilum* (*n* = 61).** The peaks were automatically identified by *Pick Peaking* procedure (Opus software).

Similarities between ECM fungi were identified for the structural carbohydrates such as β-1, 3-glucans (i.e., the shoulder band at 1078–1073 cm^−1^, slightly deviated to 1090 cm^−1^ in *A. rubescens* spectra, and the peak at 1032–1030 cm^−1^, (Galichet et al., 2001) and for chitin (i.e., the peaks at 1373 cm^−1^, 1154 cm^−1^, and 1003 cm^−1^, Wu et al., 2005, Figure 3). The infrared absorption in the 1015 to 800 cm^−1^ region revealed several bands assigned to mannans (Galichet et al., 2001; Szeghalmi et al., 2006). The ascomycete *C. geophilum* showed a specific peak at 831 cm^−1^ and lacked the shoulders to 990 cm^−1^ and 976 cm^−1^ found in other ECM spectra (Figure 3). Moreover, a particular peak at 1108 cm^−1^, associated with the vibration of the C-O bonds in polysaccharides (Szeghalmi et al., 2006), appeared in *C. geophilum* spectra (Figure 3). These differences support the high HCA heterogeneity between *C. geophilum* and other ECMs.

Storage carbohydrates such as glycogen participated in the spectral output by the shoulders at 1153 cm^−1^ in *L. subdulcis*, *R. ochroleuca* and *C. geophilum* and at 1161 cm^−1^ in *X. pruinatus* and *A. rubescens* (Figure 3). These wavenumbers were ascribed to stretching vibrations of hydrogen-bonded C-O groups (Wong et al., 1991).

The spectral profile of lipids showed two bands shared by all investigated ECM fungal taxa: the typical CH_2_ stretching of the methylene chains in lipids at 2925 cm^−1^ (Szeghalmi et al., 2006) and the CH_2_ and CH_3_ asymmetric bending vibrations in lipids at 1451 cm^−1^ (Meade et al., 2010). The peak near 1370 cm^−1^, derived from symmetric stretching of carboxylate or the CH_2_ and CH_3_ deformations of lipids (Meade et al., 2010), was common to all but *X. pruinatus* ECMs (Figure 3), while the bands at 2850 cm^−1^, assigned to CH_2_ symmetric vibrations and at 1740 cm^−1^ associated with C=O stretching in lipids (Wong et al., 1991), were present only in the spectra of *Russulaceae* species.

The spectral regions, amide I and amide II, were used for characterization of the protein component of ECMs. In *L. subdulcis*, *X. pruinatus*, and *A. rubescens* ECMs, the maximum peak of amide I, assigned to β-sheet protein structure (Kong and Yu, 2007), was found at 1632, 1630, and 1633 cm^−1^, respectively (Figure 3). In *C. geophilum* ECMs, the maximum peak was shifted to 1617 cm^−1^ while in *R. ochroleuca* two β-sheet structure-assigned peaks appeared at 1627 and 1618 cm^−1^ (Figure 3). In *R. ochroleuca* ECMs, an extra peak at 1649 cm^−1^ associated with the random coil protein structure (Kong and Yu, 2007) was observed (Figure 3).

Comparative analysis of the amide II region (1480–1575 cm^−1^) showed pronounced differences among ECMs (Figure 3); they displayed only one common peak around 1515 cm^−1^ (Figure 3) that was associated with an aromatic tyrosine ring (Meade et al., 2010). It is interesting to note that in the 1550–1540 cm^−1^ range ascribed to α-helix structures, *X. pruinatu*s and *A. rubescens* displayed two prominent identical peaks (Figure 3).

### The spectral response to environmental conditions varied with the fungal species identity

To determine whether different environments lead to different ECM intra-specific biochemical responses, we compared the mean spectra of samples, which were either collected in the mono-specific or in the mixed forest stands. ECM spectral profiles revealed a fungal species-specific response to the alteration of the environment. Because the intra-specific spectral similarity was high, we amended the spectra by Fourier self-deconvolution (FSD) technique to enhance the resolution.

**Figure 4:**
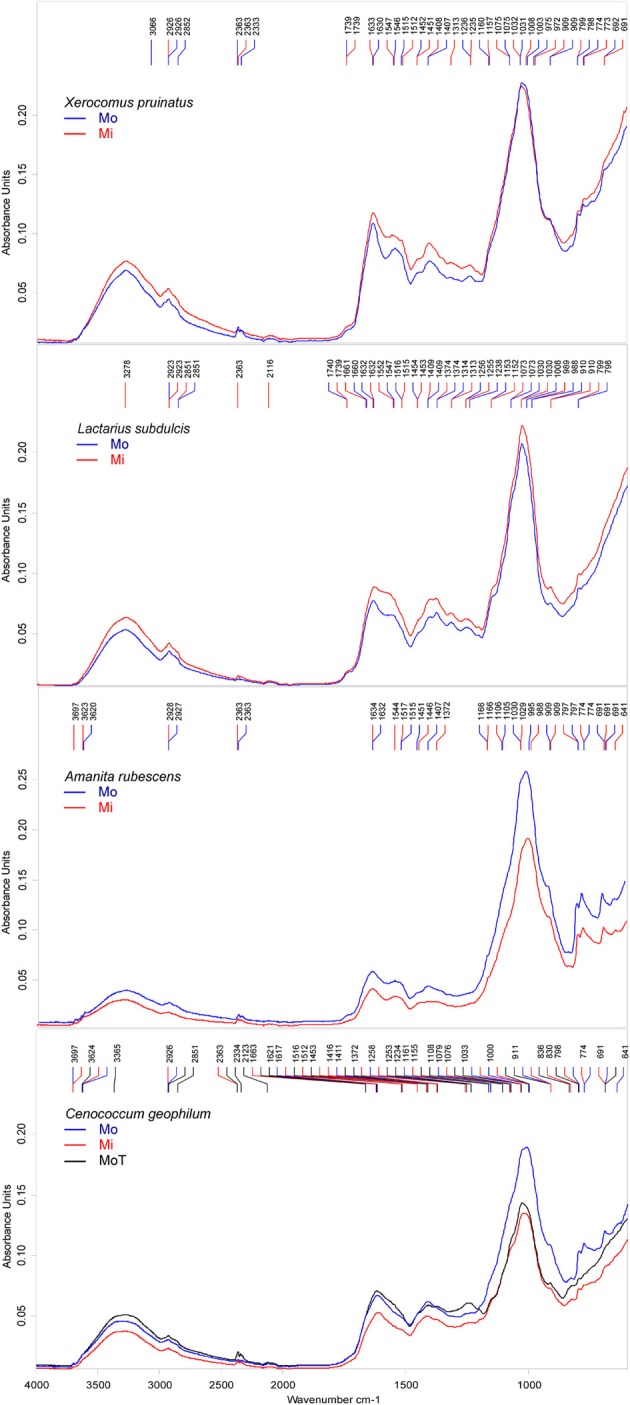
**Deconvoluted FTIR spectra of ectomycorrhizas collected in mono-specific beech (Mo) and in mixed deciduous forests (Mi) in the Hainich.** *Cenococcum geophilum* ectomycorrhiza were collected in beech forests on the Swabian Jura (T). The peaks were automatically identified by *Pick Peaking* procedure (Opus software). Each spectrum represents the average of *n* = 10 measurements; *n* = 2 for *Xerocomus pruinatus* Mo.

Major differences between the samples collected in the two different forest types were seen in protein and lipid peaks. Supplementary peaks, associated with CH_3_ and CH_2_ asymmetric bending variations in proteins, were identified in mixed forest samples of *C. geophilum* at 1372 and 1453 cm^−1^, *X. pruinatus* at 1313 cm^−1^, and *A. rubescens* at 1372 cm^−1^. Interestingly, a clear influence of the environmental conditions on the spectral profile of ECMs formed with *L. subdulcis* could not be identified in this analysis (Figure 4). However, the forest types resulted in noticeable effects on *C. geophilum* ECM spectra with five peaks at 2363, 1453, 1663, 830, and 1372 cm^−1^ in samples from the mixed stand, which were completely missing in the samples collected in beech-dominated stands (Figure 4).

*Amanita rubescens* ECMs exhibited also a particular environmentally induced spectral pattern with two peaks at 1544 and 1372 cm^−1^ that appeared in the mixed but not in the beech-dominated forest collected samples (Figure 4) and a peak at 1407 cm^−1^ that was present only in the latter samples.

Comparison of the spectra of *C. geophilum* ECMs harvested in different locations also supported that the environment variations influenced the biochemical profiles of ECMs, but with only small variations. All major *Cenococcum*-specific features that were exhibited by spectra of samples collected in different forest types in the Hainich occurred in samples harvested on the Swabian Jura (Figure 4). One minimal difference was the presence of the bands at 1621 cm^−1^ possibly assigned to protein β-sheet structure, and at 1234 cm^−1^ associated with asymmetric P-H stretching.

## Discussion

### FTIR spectroscopy discriminate ECM fungal species associated with root tips

A major goal of this work was to determine whether the FTIR signatures discriminated different ECM fungal species in the same and/or in different environments. Naumann (2000) stated that a “notable disadvantage” of FTIR spectroscopy, when it is applied to biological samples, is the necessity to use organisms cultivated under strictly controlled conditions. Here, we reject this concept for ECM fungi because we found characteristic specific variation in the spectral regions of the major biochemical constituents including carbohydrates, fatty acids, and proteins, in environmental samples of the ECMs from different sites.

Concerning the carbohydrate component, our findings support the general agreement that in many groups of fungi, the structural carbohydrates, glucans, which constitute together with the chitin the central core of the cell wall, are similar among species, while the cell wall decorating carbohydrates, covalently bound to the glucan-chitin complex such as mannans, may vary with taxon identity (Latgé, 2010; Leal et al., 2010). None of ECMs studied revealed spectral differences in bands associated with β-1, 3-glucans; yet, they showed major variation in the region of tentatively mannan-assigned bands (1015–800 cm^−1^, Galichet et al., 2001; Szeghalmi et al., 2006). Mannans are also components of the cementing layer that embeds the hyphae within the mantle of ECMs (Balestrini et al., 1996) and, therefore, may occur in sufficient concentrations to be detectable by FTIR.

Fatty acid composition has often been used in fungal taxonomy (Guarro et al., 1999). Contribution of fatty acid-associated bands to spectral discrimination between ECMs corroborates the idea of Karliński et al. (2007), who suggested that the ECM fatty acid composition varies with fungal species identity. To date, the fatty acid biosynthetic pathways have only reconstructed for ECM fungus *Laccaria bicolor* (Reich et al., 2009) and therefore, the significance of fatty acid patterns as biomarker for ECM species discrimination is still unclear.

The spectral shape in the amide I and II regions, associated with protein secondary structures revealed differences between ECM fungal species. Since ECM fungi are characterized by a high degree of functional diversity, differences in the fungal protein pattern might be related to specific functions (Kim et al., 2007). If this was the case, the information on protein secondary structures provided by FTIR spectra (Barth and Zscherp, 2002; Kong and Yu, 2007) may enhance the understanding of fungal physiology. The spectral analyses revealed a shift of the 1630 cm^−1^ peak to 1617 cm^−1^ that divided the ECMs in two groups, the group of *L. subdulcis*, *X.pruinatus*, and *A. rubescens* ECMs and the group of C*. geophilum* and *R. ochroleuca*. It is interesting to note that the fungi in latter group share the same habitat and have the same strategy for nutrient mobilization (Courty et al., 2005). The amide II region, frequently used to follow protein conformational dynamics (Kong and Yu, 2007), displayed two prominent identical peaks in *X. pruinatu*s and *A. rubescens*, which separated these from the other studied ECMs. Both species have been described as long-distance exploration type ECMs (Agerer, 2001), whose rhizomorphs are specialized for access and transport of nutrients from distant areas. In addition to ECM species separation, the current results open the interesting possibility that the specific discrimination might contain information on fungal traits.

### Environmental variation does not override species-specific FTIR spectral information

A key finding of this study was that environment variability neither limited the discrimination between ECMs fungal species by HCA nor provided sufficient resolution to discern species-associated sub-clusters for different sites. The information-rich FTIR vibrational spectra of biological samples have been proposed to be suitable indicators of structural and physiological changes associated with environmental factors (Szeghalmi et al., 2006). However, in our study the micro-climatic heterogeneity and the differences in soil structure and biochemistry caused by tree species diversity (Guckland et al., 2010) resulted only in small alterations of ECM biochemical profiles. A possible explanation for this observation is that the ECM fungi differ in their preference for substrates and exhibit niche partitioning, which may result in specific patterns of resource utilization (Dickie et al., 2002; Lindahl et al., 2007). In all species but *Lactarius*, the presence of CH_3_ and CH_2_ asymmetric bending variations in proteins was increased in samples collected in mixed forest stands. We speculate that this potential change in the protein pattern might have been caused by changes in the surface proteome because ECMs produce extracellular hydrolytic enzymes (Buée et al., 2005) whose composition varies with the substrate quality (Courty et al., 2005). A higher microbial biomass and mineralization rate causing larger leaf litter nitrogen input to the soil in the mixed compared with beech-dominated forest stands (Guckland et al., 2010) may sustain the differences in ECM enzymatic components between the two forest types (Conn and Dighton, 2000).

Interestingly, the results show species-characteristic spectral responses to different forest environments. *L. subdulcis* ECMs revealed no spectral alterations between mono-specific and mixed forests. A possible explanation might be that *L. subdulcis* ECMs independently of the environment always occupies the same niche, namely leaf litter. *L. subdulcis* has a smooth mantle and no emanating hyphae (Agerer, 2001) and, therefore, can only access nutrients in its immediate vicinity. *L. subdulcis* secrets laccase and leucine aminopeptidases, which enable the ECMs to degrade recalcitrant phenolic compounds and proteins (Courty et al., 2005; Rineau and Garbaye, 2009), while many other ECMs do not have such competences. *Amanita rubescens* and *Xerocomus pruinatus* form long-distance exploration type ECM (Agerer, 2001), which spread long rhizomorphs into the surrounding soil accessing a wide area (Kjøller, 2006). These ECM fungi experience strong soil physicochemical heterogeneity within their range (Bell and Lechowicz, 1991; Toljander et al., 2006) and, thus are probably less niche selective than *L. subdulcis*. The ubiquitous fungus *C. geophilum* (Jany et al., 2002) forms ECMs in different substrates such as organic soil, mineral soil, and dead wood debris (Buée et al., 2007; Pena et al., 2013) and therefore may have a wider nutrient range than *L. subdulcis*.

In summary, currently, we can only speculate about the reasons why some ECM species show and others do not show differences in their FTIR spectra when sampled in different environments. However, it is suspicious that the ECM species confined to leaf litter did not reveal environmental signatures whereas the other fungi with a wider range of environments exhibit small species-specific differences. Further studies are required to elucidate the relationship between habitat and fungal traits. Overall, our study indicates that FTIR spectroscopy followed by HCA is a suitable tool to discriminate between *in situ* collected ECMs. FTIR spectroscopy has clear advantages compared with other identification procedures with regard to cost efficiency and the possibility to rapidly screen large numbers of samples. The FTIR spectral signatures furthermore may enable us to gather physiological and functional *in situ* information about ECM fungi and their role for host plant performance.

### Conflict of interest statement

The authors declare that the research was conducted in the absence of any commercial or financial relationships that could be construed as a potential conflict of interest.

## References

[B1] Agerer R. (1987). Colour Atlas of Ectomycorrhizae: With Glossary. Schwäbisch Gmünd: Einhorn-Verlag

[B2] Agerer R. (2001). Exploration types of ectomycorrhizae. Mycorrhiza 11, 107–114 10.1007/s005720100108

[B3] Balestrini R. Hahn M. G. Faccio A. Mendgen K. Bonfante P. (1996). Differential localization of carbohydrate epitopes in plant cell walls in the presence and absence of arbuscular Mycorrhizal Fungi. Plant Physiol. 111, 203–213 10.1104/pp.111.1.203 12226286 PMC157827

[B4] Barth A. Zscherp C. (2002). What vibrations tell about proteins. Q. Rev. Biophys. 35, 369–430 10.1017/S0033583502003815 12621861

[B5] Bell G. Lechowicz M. J. (1991). The ecology and genetics of fitness in forest plants. I. Environmental heterogeneity measured by explant trials. J. Ecol. 79, 663–685 10.2307/2260660

[B6] Buée M. Courty P. E. Mignot D. Garbaye J. (2007). Soil niche effect on species diversity and catabolic activities in an ectomycorrhizal fungal community. Soil Biol. Biochem. 39, 1947–1955 10.1016/j.soilbio.2007.02.016

[B7] Buée M. Vairelles D. Garbaye J. (2005). Year-round monitoring of diversity and potential metabolic activity of the ectomycorrhizal community in a beech (*Fagus sylvatica*) forest subjected to two thinning regimes. Mycorrhiza 15, 235–245 10.1007/s00572-004-0313-6 15221576

[B8] Calderon F. J. Acosta-Martinez V. Douds D. D. Reeves J. B. Vigil M. F. (2009). Mid-Infrared and near-infrared spectral properties of mycorrhizal and non-mycorrhizal root cultures. Appl. Spectrosc. 63, 494–500 10.1366/000370209788346931 19470204

[B9] Conn C. Dighton J. (2000). Litter quality influences on decomposition, ectomycorrhizal community structure and mycorrhizal root surface acid phosphatase activity. Soil Biol. Biochem. 32, 489–496 10.1016/S0038-0717(99)00178-9

[B10] Courty P.-E. Pritsch K. Schloter M. Hartmann A. Garbaye J. (2005). Activity profiling of ectomycorrhiza communities in two forest soils using multiple enzymatic tests. New Phytol. 167, 309–319 10.1111/j.1469-8137.2005.01401.x 15948852

[B11] Dannenmann M. Gasche R. Papen H. (2007). Nitrogen turnover and N_2_O production in the forest floor of beech stands as influenced by forest management. J. Plant Nutr. Soil Sci. 170, 134–144 10.1002/jpln.200620644

[B12] Dannenmann M. Simon J. Gasche R. Holst J. Naumann P. S. Kögel-Knabner I. (2009). Tree girdling provides insight on the role of labile carbon in nitrogen partitioning between soil microorganisms and adult European beech. Soil Biol. Biochem. 41, 1622–1631 10.1016/j.soilbio.2009.04.024

[B13] Dickie I. A. Xu B. Koide R. T. (2002). Vertical niche differentiation of ectomycorrhizal hyphae in soil as shown by T-RFLP analysis. New Phytol. 156, 527–535 10.1046/j.1469-8137.2002.00535.x 33873568

[B14] Erukhimovitch V. Tsor L. Hazanovsky M. Talyshinsky M. Mukmanov I. Souprun Y. (2005). Identification of fungal phyto-pathogens by Fourier-transform infrared (FTIR) microscopy. J. Agric. Technol. 1, 145–152

[B15] Finlay R. D. (2008). Ecological aspects of mycorrhizal symbiosis: with special emphasis on the functional diversity of interactions involving the extraradical mycelium. J. Exp. Bot. 59, 1115–1126 10.1093/jxb/ern059 18349054

[B16] Förster J. Famili I. Fu P. Palsson B. Ø. Nielsen J. (2003). Genome-scale reconstruction of the *Saccharomyces cerevisiae* metabolic network. Genome Res. 13, 244–253 10.1101/gr.234503 12566402 PMC420374

[B17] Galichet A. Sockalingum G. D. Belarbi A. Manfait M. (2001). FTIR spectroscopic analysis of *Saccharomyces cerevisiae* cell walls: study of an anomalous strain exhibiting a pink-colored cell phenotype. FEMS Microbiol. Lett. 197, 179–186 10.1111/j.1574-6968.2001.tb10601.x 11313132

[B58] Griffiths P. R. Haseth J. A. (2007). Fourier Transform Infrared Spectroscopy. New Jersey, NJ: John Wiley & Sons, Inc

[B18] Guarro J. Gené J. Stchigel A. M. (1999). Developments in fungal taxonomy. Clin. Microbiol. Rev. 12, 454–500 10398676 10.1128/cmr.12.3.454 PMC100249

[B19] Guckland A. Corre M. D. Flessa H. (2010). Variability of soil N cycling and N_2_O emission in a mixed deciduous forest with different abundance of beech. Plant Soil 336, 25–38 10.1007/s11104-010-0437-8

[B20] Guckland A. Jacob M. Flessa H. Thomas F. M. Leuschner C. (2009). Acidity, nutrient stocks, and organic-matter content in soils of a temperate deciduous forest with different abundance of European beech (*Fagus sylvatica* L.). J. Plant Nutr. Soil Sci. 172, 500–511 10.1002/jpln.200800072

[B21] Jacob M. Viedenz K. Polle A. Thomas F. M. (2010). Leaf litter decomposition in temperate deciduous forest stands with a decreasing fraction of beech (*Fagus sylvatica*). Oecologia 164, 1083–1094 10.1007/s00442-010-1699-9 20596729 PMC2981742

[B22] Jany J.-L. Garbaye J. Martin F. (2002). *Cenococcum geophilum* populations show a high degree of genetic diversity in beech forests. New Phytol. 154, 651–659 10.1046/j.1469-8137.2002.00408.x 33873469

[B23] Karliński L. Ravnskov S. Kieliszewska-Rokicka B. Larsen J. (2007). Fatty acid composition of various ectomycorrhizal fungi and ectomycorrhizas of Norway spruce. Soil Biol. Biochem. 39, 854–866 10.1016/j.soilbio.2006.10.003

[B24] Kim Y. Nandakumar M. P. Marten M. R. (2007). Proteomics of filamentous fungi. Trends Biotechnol. 25, 395–400 10.1016/j.tibtech.2007.07.008 17681627

[B25] Kjøller R. (2006). Disproportionate abundance between ectomycorrhizal root tips and their associated mycelia. FEMS Microbiol. Ecol. 58, 214–224 10.1111/j.1574-6941.2006.00166.x 17064263

[B26] Kong J. Yu S. (2007). Fourier transform infrared spectroscopic analysis of protein secondary structures. Acta. Biochim. Biophys. Sin. 39, 549–559 10.1111/j.1745-7270.2007.00320.x 17687489

[B27] Krämer I. Hölscher D. (2010). Soil water dynamics along a tree diversity gradient in a deciduous forest in Central Germany. Ecohydrology 3, 262–271 10.1002/eco.103

[B28] Lang C. Polle A. (2011). Ectomycorrhizal fungal diversity, tree diversity and root nutrient relations in a mixed Central European forest. Tree Physiol. 31, 531–538 10.1093/treephys/tpr042 21636693

[B29] Lang C. Seven J. Polle A. (2011). Host preferences and differential contributions of deciduous tree species shape mycorrhizal species richness in a mixed Central European forest. Mycorrhiza 21, 297–308 10.1007/s00572-010-0338-y 20886243 PMC3077745

[B30] Latgé J.-P. (2010). Tasting the fungal cell wall. Cell. Microbiol. 12, 863–872 10.1111/j.1462-5822.2010.01474.x 20482553

[B31] Leal J. A. Prieto A. Bernabé M. Hawksworth D. L. (2010). An assessment of fungal wall heteromannans as a phylogenetically informative character in ascomycetes. FEMS Microbiol. Rev. 34, 986–1014 10.1111/j.1574-6976.2010.00225.x 20491931

[B32] Leuschner C. Jungkunst H. F. Fleck S. (2009). Functional role of forest diversity: pros and cons of synthetic stands and across-site comparisons in established forests. Basic Appl. Ecol. 10, 1–9 10.1016/j.baae.2008.06.001

[B33] Lindahl B. D. Ihrmark K. Boberg J. Trumbore S. E. Högberg P. Stenlid J. (2007). Spatial separation of litter decomposition and mycorrhizal nitrogen uptake in a boreal forest. New Phytol. 173, 611–620 10.1111/j.1469-8137.2006.01936.x 17244056

[B34] Linker R. Tsror Lahkim L. (2008). Discrimination of soil-borne fungi using Fourier transform infrared attenuated total reflection spectroscopy. Appl. Spectrosc. 62, 302–305 10.1366/000370208783759678 18339238

[B35] Meade A. D. Clarke C. Byrne H. J. Lyng F. M. (2010). Fourier transform infrared microspectroscopy and multivariate methods for radiobiological dosimetry. Radiat. Res. 173, 225–237 10.1667/RR1836.1 20095855

[B36] Mohaček-Grošev V. Božac R. Puppels G. J. (2001). Vibrational spectroscopic characterization of wild growing mushrooms and toadstools. Spectrochim. Acta. A. Mol. Biomol. Spectrosc. 57, 2815–2829 10.1016/S1386-1425(01)00584-4 11789883

[B37] Mölder A. Bernhardt-Römermann M. Schmidt W. (2008). Herb-layer diversity in deciduous forests: raised by tree richness or beaten by beech? For. Ecol. Manag. 256, 272–281 10.1016/j.foreco.2008.04.012

[B38] Naumann A. (2009). A novel procedure for strain classification of fungal mycelium by cluster and artificial neural network analysis of Fourier transform infrared (FTIR) spectra. Analyst 134, 1215–1223 10.1039/b821286d 19475151

[B39] Naumann A. Heine G. Rauber R. (2010). Efficient discrimination of oat and pea roots by cluster analysis of Fourier transform infrared (FTIR) spectra. Field Crops Res. 119, 78–84 10.1016/j.fcr.2010.06.017

[B40] Naumann A. Navarro-González M. Peddireddi S. Kües U. Polle A. (2005). Fourier transform infrared microscopy and imaging: detection of fungi in wood. Fungal Genet. Biol. 42, 829–835 10.1016/j.fgb.2005.06.003 16098775

[B41] Naumann D. (2000). Infrared spectroscopy in microbiology, in Encyclopedia of Analytical Chemistry, ed. Meyers R. A. (New Jearsy, NJ: John Wiley & Sons Ltd.), 102–131

[B42] Pena R. Offermann C. Simon J. Naumann P. S. Gessler A. Holst J. Dannenmann M. (2010). Girdling affects ectomycorrhizal fungal (EMF) diversity and reveals functional differences in EMF community composition in a beech forest. Appl. Environ. Microbiol. 76, 1831–1841 10.1128/AEM.01703-09 20097809 PMC2837996

[B43] Pena R. Polle A. (2014). Attributing functions to ectomycorrhizal fungal identities in assemblages for nitrogen acquisition under stress. ISME J. 8, 321–330 10.1038/ismej.2013.158 24030593 PMC3906819

[B44] Pena R. Tejedor J. Zeller B. Dannenmann M. Polle A. (2013). Interspecific temporal and spatial differences in the acquisition of litter-derived nitrogen by ectomycorrhizal fungal assemblages. New Phytol. 199, 520–528 10.1111/nph.12272 23594339

[B45] Reich M. Göbel C. Kohler A. Buée M. Martin F. Feussner I. (2009). Fatty acid metabolism in the ectomycorrhizal fungus Laccaria bicolor. New Phytol. 182, 950–964 10.1111/j.1469-8137.2009.02819.x 19383096

[B46] Rineau F. Garbaye J. (2009). Does forest liming impact the enzymatic profiles of ectomycorrhizal communities through specialized fungal symbionts? Mycorrhiza 19, 493–500 10.1007/s00572-009-0249-y 19421790

[B47] Saitou N. Nei M. (1987). The neighbor-joining method: a new method for reconstructing phylogenetic trees. Mol. Biol. Evol. 4, 406–425 3447015 10.1093/oxfordjournals.molbev.a040454

[B48] Schützendübel A. Polle A. (2002). Plant responses to abiotic stresses: heavy metal-induced oxidative stress and protection by mycorrhization. J. Exp. Bot. 53, 1351–1365 10.1093/jexbot/53.372.1351 11997381

[B59] Simon J. Dannenmann M. Gasche R. Holst J. Mayer H. Papen H. (2011). Competition for nitrogen between adult European beech and its offspring is reduced by avoidance strategy. For. Ecol. Manage. 262, 105–114 10.1016/j.foreco.2011.01.035

[B49] Smith S. E. Read D. J. (2008). Mycorrhizal Symbiosis. New York, NY: Academic Press

[B50] Suz L. M. Azul A. M. Morris M. H. Bledsoe C. S. Martín M. P. (2008). Morphotyping and molecular methods to characterize ectomycorrhizal roots and hyphae in soil, in Molecular Mechanisms of Plant and Microbe Coexistence Soil Biology, eds Nautiyal P. D. C. S. Dion P. D. P. (Berlin, Heidelberg: Springer), 437–474

[B51] Szeghalmi A. Kaminskyj S. Gough K. M. (2006). A synchrotron FTIR microspectroscopy investigation of fungal hyphae grown under optimal and stressed conditions. Anal. Bioanal. Chem. 387, 1779–1789 10.1007/s00216-006-0850-2 17106657

[B52] Tamura K. Kumar S. (2002). Evolutionary distance estimation under heterogeneous substitution pattern among lineages. Mol. Biol. Evol. 19, 1727–1736 10.1093/oxfordjournals.molbev.a003995 12270899

[B53] Tamura K. Peterson D. Peterson N. Stecher G. Nei M. Kumar S. (2011). MEGA5: molecular evolutionary genetics analysis using maximum likelihood, evolutionary distance, and maximum parsimony methods. Mol. Biol. Evol. 28, 2731–2739 10.1093/molbev/msr121 21546353 PMC3203626

[B54] Toljander J. F. Eberhardt U. Toljander Y. K. Paul L. R. Taylor A. F. S. (2006). Species composition of an ectomycorrhizal fungal community along a local nutrient gradient in a boreal forest. New Phytol. 170, 873–884 10.1111/j.1469-8137.2006.01718.x 16684245

[B55] Wilson R. H. Smith A. C. Kačuráková M. Saunders P. K. Wellner N. Waldron K. W. (2000). The mechanical properties and molecular dynamics of plant cell wall polysaccharides studied by Fourier-transform infrared spectroscopy. Plant Physiol. 124, 397–406 10.1104/pp.124.1.397 10982452 PMC59152

[B56] Wong P. T. Wong R. K. Caputo T. A. Godwin T. A. Rigas B. (1991). Infrared spectroscopy of exfoliated human cervical cells: evidence of extensive structural changes during carcinogenesis. Proc. Natl. Acad. Sci. U.S.A. 88, 10988–10992 1763013 10.1073/pnas.88.24.10988 PMC53058

[B57] World Reference Base for Soil Resources. (2006). A Framework for International Classification, Correlation and Communication (2006). Rome: Food and Agriculture Org

[B60] Wu T. Zivanovic S. Draughon F. A. Conway W. S. Sams C. E. (2005). Physicochemical properties and bioactivity of fungal chitin and chitosan. J. Agric. Food Chem. 53, 3888–3894 10.1021/jf048202s 15884813

