# Supplementary material for: Ectomycorrhizal identification in environmental samples of tree roots by Fourier-transform infrared (FTIR) spectroscopy
Source: Front Plant Sci. 2014 May 27;5:229. doi: 10.3389/fpls.2014.00229 (PMC4034152; doi:10.3389/fpls.2014.00229)
Supplement: Supplementary file 1 [file DataSheet1.DOCX]

***Supplementary Material***

**Ectomycorrhizal identification in environmental samples of tree roots by Fourier-transform infrared (FTIR) spectroscopy**

**Rodica Pena^1^*, Christa Lang^1,2^, Andrea Polle^1^**

^1^ Forest Botany and Tree Physiology, Büsgen-Institut, Georg-August University Göttingen, Göttingen, Germany

^2^ present and permanent address: Hochschule Rhein-Waal, Kamp-Lintfort, Germany

*** Correspondence:** Rodica Pena, Forest Botany and Tree Physiology, Büsgen-Institut, Georg-August University Göttingen, Büsgenweg 2, 37077, Göttingen, Germany

rpena@gwdg.de

1. **Supplementary Figures and Tables**

## Supplementary Tables

**Supplementary Table 1.** Description of ectomycorrhizas. The morphotypes were identified by internal transcribed spacer (ITS) sequencing; the sequences were deposited in the NCBI GenBank under the accession numbers specified below. Bar 500 µm.

| Fungal taxa | Photo | [Organisation](http://www.deemy.de/Descriptors/CharacterDefinition.cfm?CID=76&CharID=66) of outer mantle* |
| --- | --- | --- |
| [*Cenococcum geophilum*](http://www.deemy.de/Taxa/ItemDescription.cfm?ItemID=30)  EU346870  Exploration type: short distance | 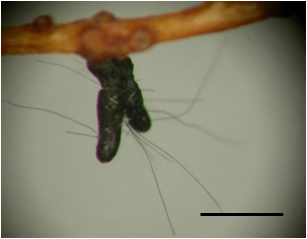 | Plectenchymatous  Hyphae star-like arranged and tightly glued together (type G)  Cell diameter: 2-15µm |
| *Xerocomus pruinatus*  EU350582  Exploration type: long distance | 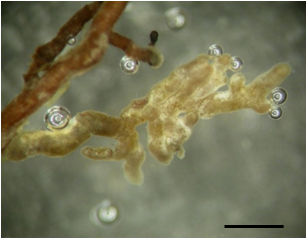 | Plectenchymatous  Ring-like arrangement of hyphal bundles (type A)  Cell diameter: 3-12µm |
| *Amanita rubescens*  EU346872  Exploration type: long distance | 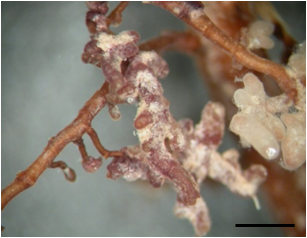 | Plectenchymatous  Ring-like arrangement of hyphal bundles (type A)  Tannin cells present in the mantle |
| *Lactarius subdulcis*  EU346875  Exploration type: contact | 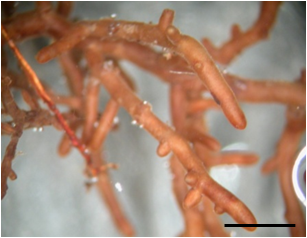 | Pseudoparenchymatous  Angular cells bearing a hyphal net  (type P)  Laticifers present in the mantle  Cell diameter: 2.5-10µm |
| *Russula ochroleuca*  EU350580  Exploration type: contact | 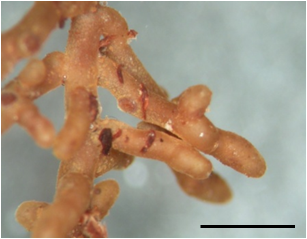 | Pseudoparenchymatous  Angular cells and mounds of flattened cells (type O)  Cell diameter: 4-20µm |

* description after DEEMY database: [www.deemy.de](http://www.deemy.de)

## Supplementary Figures


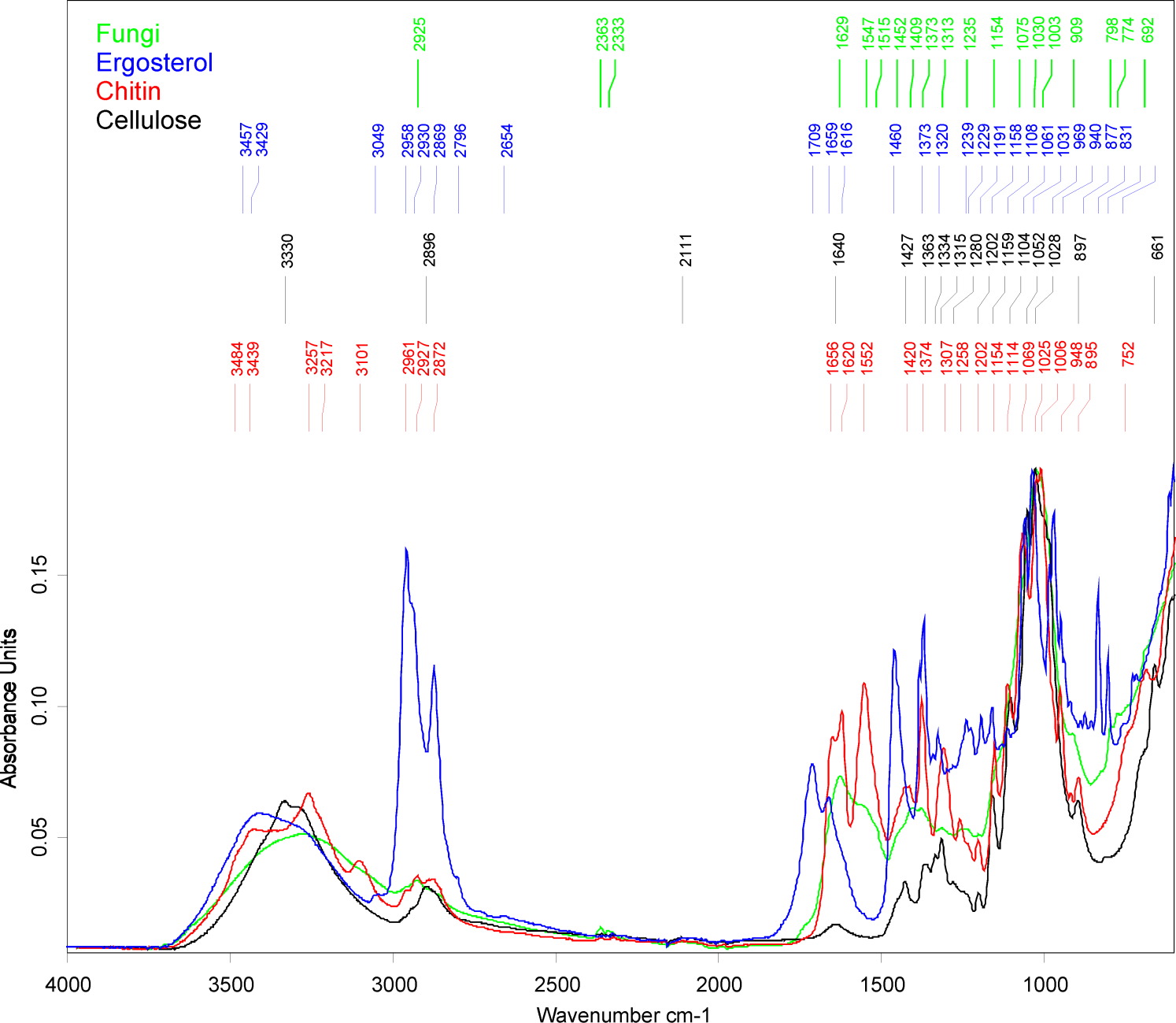


Supplementary Figure 1. FTIR spectra of field-collected ectomycorrhizas, and pure erosterol, chitin and cellulose. The peaks were automatically identified by *Pick Peaking* procedure (Opus software). The spectra represents the mean of n=181 measurements for ectomycorrhizas and n=3 measurements for pure substances.
