# Supplementary figures and images for: Ectomycorrhizal identification in environmental samples of tree roots by Fourier-transform infrared (FTIR) spectroscopy
Source: Front Plant Sci. 2014 May 27;5:229. doi: 10.3389/fpls.2014.00229 (PMC4034152; doi:10.3389/fpls.2014.00229)

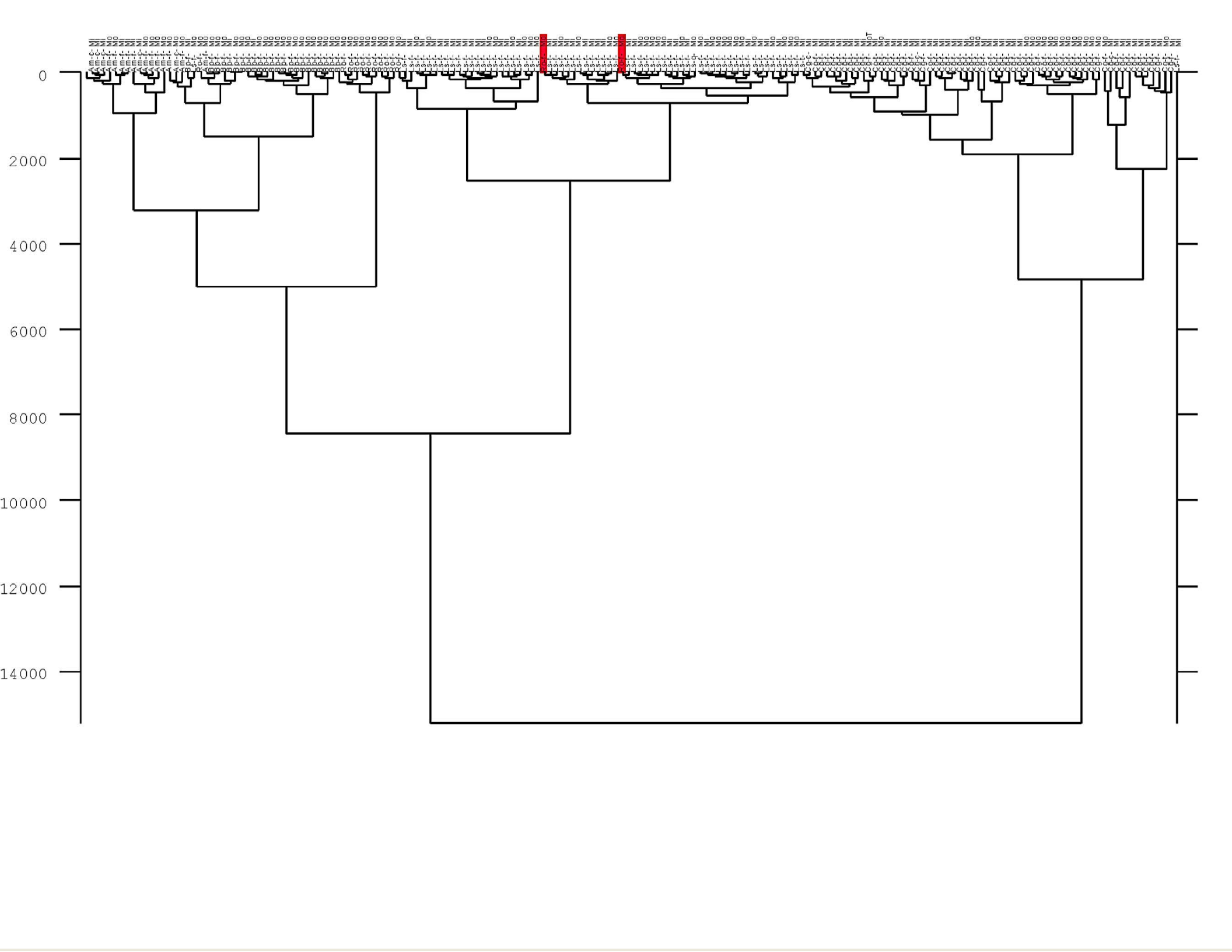

Supplement: Supplementary file 2 [file DataSheet2.PDF]
